# The Preparation of Au@TiO_2_ Yolk–Shell Nanostructure and its Applications for Degradation and Detection of Methylene Blue

**DOI:** 10.1186/s11671-017-2313-4

**Published:** 2017-09-18

**Authors:** Gengping Wan, Xiange Peng, Min Zeng, Lei Yu, Kan Wang, Xinyue Li, Guizhen Wang

**Affiliations:** Key Laboratory of Advanced Materials of Tropical Island Resources (Hainan University), Ministry of Education, Haikou, 570228 People’s Republic of China

**Keywords:** Au@TiO_2_, Yolk–shell nanostructure, Surface plasmon resonance, Photocatalysis, SERS

## Abstract

This paper reports the synthesis of a new type of Au@TiO_2_ yolk–shell nanostructures by integrating ion sputtering method with atomic layer deposition (ALD) technique and its applications as visible light-driven photocatalyst and surface-enhanced Raman spectroscopy (SERS) substrate. Both the size and amount of gold nanoparticles confined in TiO_2_ nanotubes could be facilely controlled via properly adjusting the sputtering time. The unique structure and morphology of the resulting Au@TiO_2_ samples were investigated by using various spectroscopic and microscopic techniques in detail. It is found that all tested samples can absorb visible light with a maximum absorption at localized surface plasmon resonance (LSPR) wavelengths (550–590 nm) which are determined by the size of gold nanoparticles. The Au@TiO_2_ yolk–shell composites were used as the photocatalyst for the degradation of methylene blue (MB). As compared with pure TiO_2_ nanotubes, Au@TiO_2_ composites exhibit improved photocatalytic properties towards the degradation of MB. The SERS effect of Au@TiO_2_ yolk–shell composites was also performed to investigate the detection sensitivity of MB.

**Electronic supplementary material:**

The online version of this article (10.1186/s11671-017-2313-4) contains supplementary material, which is available to authorized users.

## Background

Heterogeneous metal/semiconductor nanocomposites have attracted tremendous research interest by virtue of their unique physic-chemical properties and potential applications in solar energy conversion [1], biomedicine [2], surface-enhanced Raman scattering [3], light-emitting diodes [4], and environmental remediation [5]. Motivated by their various applications, a vast number of efforts have been paid to design and modulate the compositions, nanostructures, and dimensions of such materials [6–8]. For example, Yin et al. [9] synthesized ZnO/Ag and ZnO/Pd hybrid nanostructures and found that the deposition of Ag or Pd onto ZnO tremendously improved photocatalytic activity of ZnO. Sun et al. [10] demonstrated that Au-Fe_3_O_4_ nanoparticles with nanoscale interactions between Au and Fe_3_O_4_ exhibited a rich variety of magnetic, physical, and chemical properties.

In recent years, significant advances in the controlled synthesis of metal/semiconductors applied to photocatalysis have been made due to the increasingly serious environmental problems such as air pollution [11, 12] and potential technical applications in energy conversion [13]. Among the various metal/semiconductor composites that have been proposed, those involving TiO_2_ and nano Au are the most practical as such heterostructure has strong localized surface plasmon resonance (LSPR) in the visible spectrum range and makes it a new kind of wide-spectrum-response photocatalyst [14–16]. Another advantageous function of Au/TiO_2_ nanocomposites is that Au nanoparticles work as electron storage, effectively reducing the recombination of photoexcited electron-hole pairs, and eventually increasing the quantum yield of photocatalysis [17, 18]. Some innovative investigations based on Au/TiO_2_ composite system applied in degradation of organic dyes, solar water splitting, and conversion of organic compounds have demonstrated their efficient visible light photocatalytic features, indicating a crucial role of the plasmonic effects of Au played in Au/TiO_2_ system [17, 19, 20].

However, one of the main limitations for the Au/TiO_2_ nanocomposites translated into practical applications is the poor stability of the supported gold catalysts. The outstanding properties presented in the original nanoparticles may weaken as they tend to agglomerate and grow into larger particles under a variety of reaction conditions [21, 22]. And in some other cases, it has been proved that Au nanoparticles deposited on the surfaces of TiO_2_ are likely to undergo corrosion or dissolution during a catalytic reaction [23]. The design and construction of core–shell and yolk–shell structured composites are considered as an effective method to address these issues. Gong et al. [24] reported the fabrication of gold nanorod@TiO_2_ yolk–shell catalysts with different aspect ratios of gold nanorod through a seed-mediated method. The multicomponent hybrid nanocomposites also present the enhanced photocatalytic activities in the oxidation reaction of benzyl alcohol. Zaera and co-workers [21] reported on the synthesis and characterization of a new Au@TiO_2_ yolk–shell-nanostructured catalyst, showing a promoting activity comparable to those observed with more conventional Au/TiO_2_ catalysts but an improved stability against sintering. Kim et al. [25] synthesized core–shell plasmonic nanostructures consisting of Au–TiO_2_ supported on SiO_2_ spheres in dye-sensitized solar cells (DSSCs), which exhibited observably enhanced power conversion efficiencies of ~ 14%. Despite tremendous research efforts have been made, the facile synthesis of Au@TiO_2_ composites with a well-defined core–shell/yolk–shell structure still remains a challenge for mass application.

Recently, many studies confirmed that controlled chirality at the nanoscale might induce a greater LSPR effect because a multihelical chiral nanostructure can give rise to induced birefringence at the microscopic scale and generate the Kerr effect caused by an induced electric field at the macroscopic scale [26–28]. In this study, the Au@TiO_2_ yolk–shell nanocomposites with helical fiber-like structure have been successfully synthesized by a controllable and facile strategy. The gold nanoparticles loaded on the surface of carbon nanocoils (CNCs) were produced by ion sputtering. The TiO_2_ films with highly uniform and controlled thickness could be integrated steady on the surface of gold nanoparticles by an atomic layer deposition (ALD) technology. Followed by an annealing step, the Au@TiO_2_ nanocomposites were obtained. The above-developed method can also be extended to fabricate other metal (Pt, Ag)@TiO_2_ yolk–shell nanocomposites with a helical nanostructure. As a representative photocatalyst, the photocatalytic activities of obtained Au@TiO_2_ nanocomposites were evaluated by degradation of methylene blue (MB) under visible light irradiation. In addition, the surface-enhanced Raman spectroscopy (SERS) activities of Au@TiO_2_ nanocomposites were also investigated through detection of MB.

### Experimental

#### Synthesis of Au@TiO_2_

CNCs used as templates were prepared by chemical vapor deposition method as reported previously. Briefly, acetylene and copper nanoparticles were used as the carbon source and the appropriate catalysts, respectively. The growth of CNCs was carried out at atmospheric pressure in a horizontal quartz tube. A ceramic plate containing the copper catalysts was placed in the reactor. After the tube was heated to 250 °C in vacuum, acetylene was introduced into the reactor [29–31]. After the apparatus was cooled to room temperature, the as-prepared CNCs were obtained.

The obtained CNCs were dispersed in ethanol under ultrasonic stirring and then daubed uniformly on the surface of a glass slide. After being dried in ambient air, the Au layer was deposited by an ion sputtering instrument (Hitachi, E-1010). The size and thickness of Au films were determined by discharge current and sputtering time. In this step, the discharge current was 10 mA and the sputtering time varied from 30 to 120 s. The obtained samples were marked as CNCs@Au-*x*, in which *x* refers to the sputtering time (seconds). Subsequently, the samples were dispersed in ethanol by ultrasonic agitation and then spread onto a quartz wafer to be coated with TiO_2_ by ALD process. ALD is a kind of vapor-phase coating preparation technique and can achieve precise thickness control and excellent uniformity of films [32–36]. ALD process was carried out in a hot-wall, flow-type ALD reactor at 145 °C with titanium tetraisopropanolate (TTIP) and deionized H_2_O used as the titanium and oxygen precursors, respectively. Finally, after the ALD process, the above-coated nanocoils were calcined at 450 °C for 2 h in air under ambient pressure to remove the carbon cores and the helical TiO_2_-coated Au yolk–shell structures were obtained. For comparison, the pure TiO_2_ helical tube was also collected by calcinated TiO_2_-coated CNCs without sputtering Au and is denoted as TiO_2_ in the following discussion.

### Material Characterization

X-ray diffraction (XRD) patterns were recorded on a Bruker D8 Advance diffractometer with copper Kα (*λ* = 0.154056 nm) radiation source. Scanning electron microscopy (SEM) images were acquired with a Hitachi S-4800 microscope. Transmission electron microscopy (TEM), selected area electron diffraction (SAED), and high-resolution TEM (HRTEM) images were obtained using a JEOL JEM-2100 microscope instrument operated at 200 kV. X-ray photoelectron spectroscopy (XPS) data were acquired using a PHI5000 Versaprobe-II spectrometer with a monochromatic Al Kα (1486.6 eV) source. Optical absorption spectra were recorded using a PerkinElmer Lambda 750s UV–Vis–NIR absorption spectrophotometer. The Raman scattering spectra were recorded on a Renishaw Invia Reflex Laser Raman spectrometer. The excitation wavelength was 514 nm from an air-cooled argon ion laser with an effective power of 2 mW.

### Photocatalytic Activities Evaluation

The photocatalytic activities of catalysts were investigated by the photodegradation of MB dyes in aqueous solutions using the procedure as described below. Two milligrams of catalyst was spread uniformly into a 100-mL photoreactor equipped with circulating cooling water pipes. Then, 20 mL of 0.01 mg/mL MB solutions was added into the photoreactor. Before photoirradiation, the system was ultrasonically mixed for 2 min and bidirectional magnetic stirred for 30 min both in the dark in order to balance the adsorption–desorption between the photocatalysts and MB. The above 100-mL photoreactor containing suspension was then irradiated under a 300 W xenon lamp (Beijing Perfectlight Technology Co. Ltd., PLS-SXE300C) with cutoff filters so that wavelengths of light between 420 and 780 nm reached the solutions. During the process of photocatalytic reaction, the irradiation intensity was ~ 154 mW cm^−2^ and the cooling water was kept flowing to dispel thermal effect of the system. At the time intervals of every 10 min for a total time of 90 min, a portion (1 mL) of the suspensions was pipetted and immediately diluted to 3 mL, and 2 mL supernate was collected after centrifugal separation. Eventually, the residual concentration of MB in the supernate was analyzed by using an UV–Vis–NIR spectrophotometer at the solution’s characteristic wavelength (*λ*_MB_ = 664 nm).

## Results and Discussion

### Morphology and Phase Structure Analysis

**Fig. 1:**
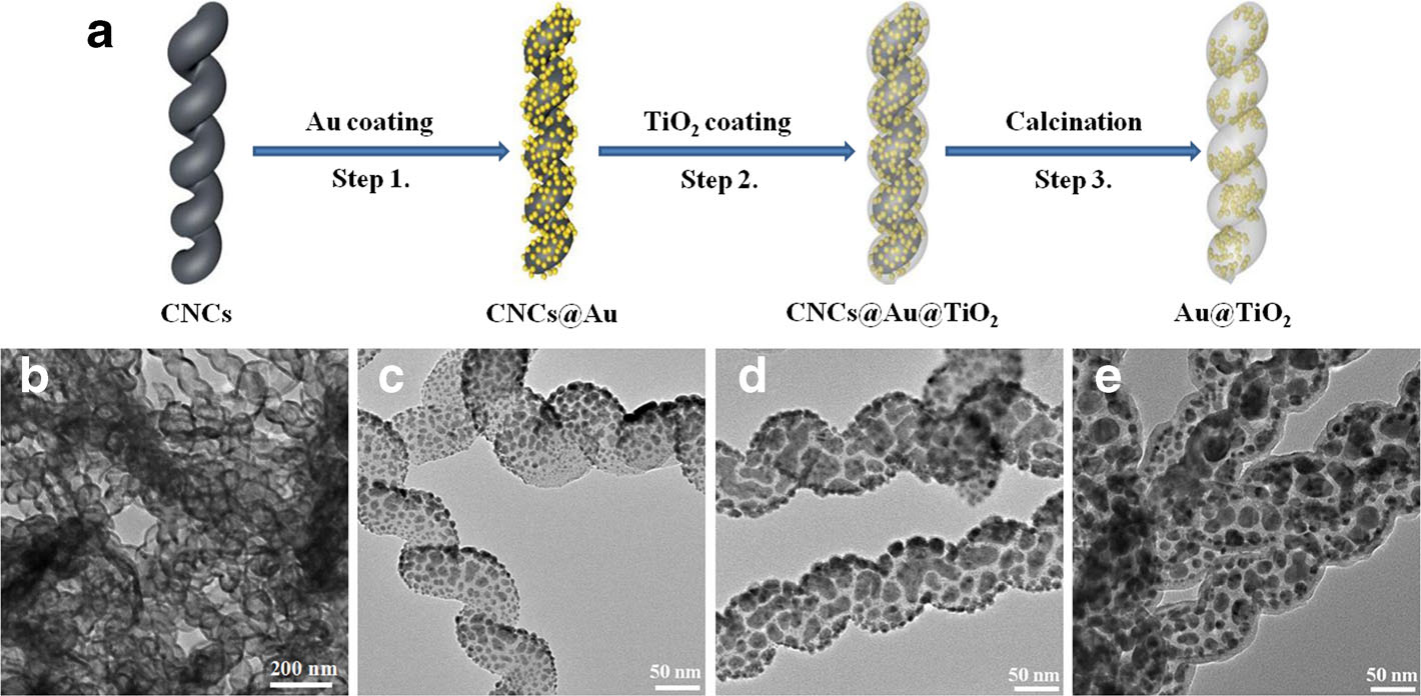
**a** Schematic illustration of the synthetic process of Au-x@TiO_2_. **b**–**e** TEM images reveal the morphological evolution

Figure 1a displays a schematic preparation flow of Au@TiO_2_ yolk–shell heterostructure, including Au sputtering, TiO_2_ coating, and calcination processes. Figure 1b–e shows typical TEM images corresponding to the above every procedure. The CNCs used as the starting template in this work have uniform fiber diameter, coil diameter, and coil pitch, and the average diameter of the fiber is about 80 nm (Additional file 1: Figure S1). After the Au sputtering treatment, the outer layer of CNCs was coated with numerous uniform Au nanoparticles as shown in Fig. 1c. As seen from the TEM image shown in Fig.1d, by applying 200 ALD cycles for TiO_2_ deposition, a uniform TiO_2_ coating with a thickness of about 8 nm is coated on the surface of Au/CNCs. Generally, the anatase phase of TiO_2_ has much better photocatalytic performance than that of rutile [37, 38]. For this reason, we chose 450 °C as a proper calcination temperature to remove the carbon cores and get the final Au@TiO_2_ yolk–shell structure. As displayed in Fig.1e, the TiO_2_ nanotubes with encapsulated Au nanoparticles and free space were formed. After all processing steps, the elegant helical morphology of the starting CNCs can be well maintained.

**Fig. 2:**
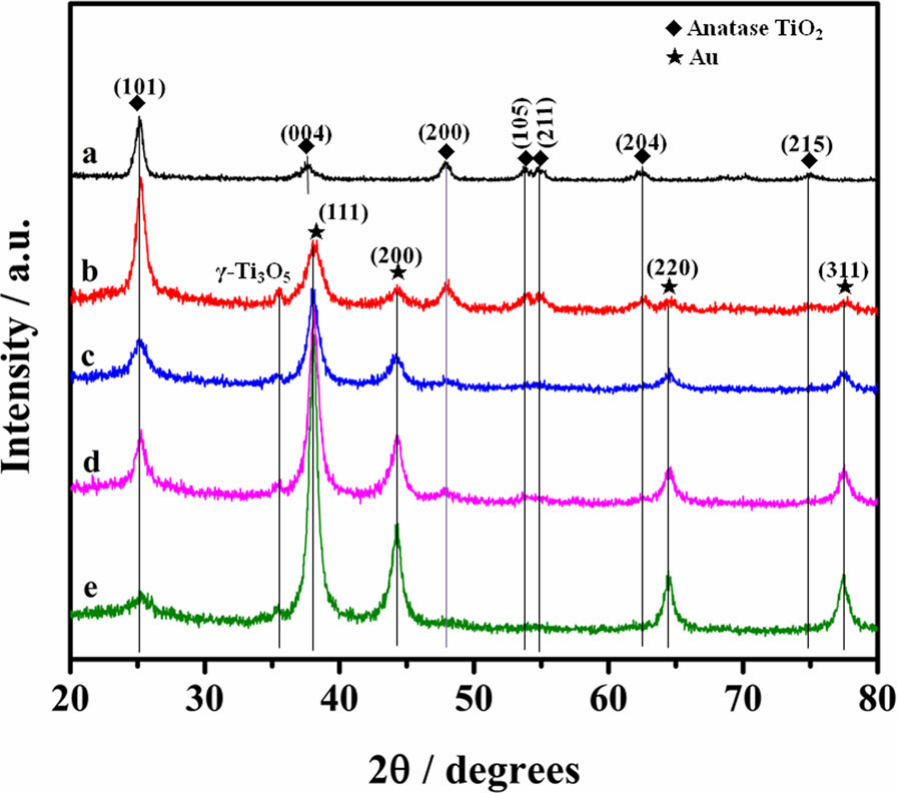
XRD patterns. a TiO_2_. b Au-30@TiO_2_. c Au-50@TiO_2_. d Au-80@TiO_2_. e Au-120@TiO_2_

The crystallinity and structures of all samples were measured by XRD. As observed in Fig. 2a, the diffraction peaks for pure TiO_2_ sample can be ascribed to well-crystallized anatase phase (JCPDS 21-1272), without additional impurity peaks. For Au/TiO_2_, the additional diffraction peaks in Fig. 2b–e can be well indexed to the face-centered cubic (FCC) Au (JCPDS 01-1174), which conformed the successful coating of Au nanoparticles on the surface of CNCs by ion sputtering. The TiO_2_ (004) peak at 38.2° has large overlap with the Au (111) peak at 38.3°. It is interesting that a weak peak located at 35.5 degrees in Fig. 2b–e can be indexed to the (020) plane of *γ*-Ti_3_O_5_, indicating that the Ti/O atomic ratio is not exactly 1/2 for Au/TiO_2_. In present work, the strong reducing action of carbon fiber and Au nanoparticles under high temperature likely induces the production of oxygen vacancies and lower oxidation states of titanium. In addition, due to the decrease of relative content for TiO_2_, it can be observed that all TiO_2_ diffraction peaks become weaker with the increased sputtering time from 30 to 120 s.

**Fig. 3:**
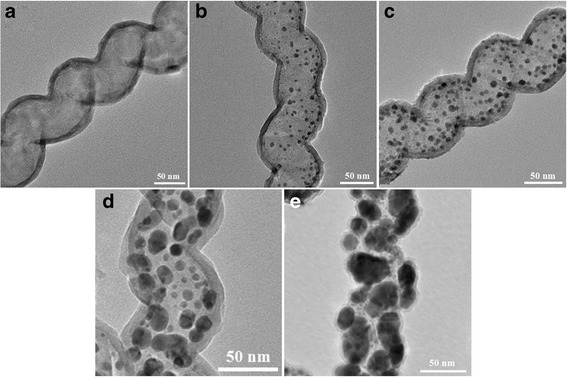
TEM images. **a** TiO_2_. **b** Au-30@TiO_2_. **c** Au-50@TiO_2_. **d** Au-80@TiO_2_. **e** Au-120@TiO_2_

Figure 3 shows the TEM images of TiO_2_ and Au-x@TiO_2_ with different Au sputtering time (*x* signifies sputtering time, *x* = 30, 50, 80, 120). For TiO_2_, it can be observed that the sample displays a helical tubular structure similar to that of the CNC templates. No collapse of the shell materials occurred during the annealing process to remove the carbon cores. The TiO_2_ shell is about 8-nm thick after 200 cycles. On account of a larger atomic number of Au compared to that of Ti in Au@TiO_2_, Au nanoparticles show a darker contrast resulting in clearly visible yolk–shell morphology. The average diameter of Au nanoparticles clearly increases with the increased sputtering time. It amounts to about 4.5, 5.5, 10.5, and 20.5 nm corresponding to the sputtering time of 30, 50, 80, and 120 s, respectively (Additional file 1: Figure S2, a2-d2). As shown in Fig. 3b–d, the homogeneous TiO_2_ thin film with about the thickness of 8 nm is also obtained for Au-30@TiO_2_, Au-50@TiO_2,_ and Au-80@TiO_2_ nanocomposites with the same ALD TiO_2_ deposition. However, the thickness of TiO_2_ shell for Au-120@TiO_2_ declines to about 5 nm (Fig. 3e), which can be ascribed to the influence of large size and significant conglomerations of Au nanoparticles.

**Fig. 4:**
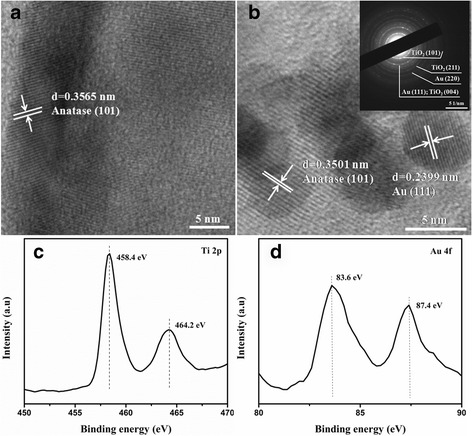
HRTEM images of **a** TiO_2_ and **b** Au-30@TiO_2_, in which the top right inset in **b** shows the SAED patterns of Au-30@TiO_2_ nanostructure. High-resolution XPS of **c** Ti 2p and **d** Au 4f of Au-30@TiO_2_

The detailed microscopic structures of the TiO_2_ and Au-30@TiO_2_ nanocompositions were further investigated by HRTEM. As observed in Fig. 4a–b, both TiO_2_ shells and Au nanoparticles are well crystallized assigned to anatase TiO_2_ (101) (0.3565 and 0.3501 nm) and Au (111) (0.2399 nm) crystalline lattices, respectively. It should be noted that the interface in Au/TiO_2_ yolk–shell nanostructures is clearly visible (Fig. 4b) because of the different contrast. Such rich interface is important for the following photocatalysis application as it may provide the access for hot electron transportation from Au nanoparticles to TiO_2_ upon LSPR excitation [20]. The inset in Fig. 4b displays the SAED pattern recorded on Au-30@TiO_2_ nanostructure. The clear diffraction rings can be attributed to (101) and (211) crystal planes of anatase TiO_2_ and (220) and (111) crystal planes of Au, respectively, in agreement with the XRD results. In order to analyze the chemical state of Au and acquire in-depth fundamental information on the interaction of Au with TiO_2_, Au-30@TiO_2_ nanocomposite was further investigated by XPS measurements. The high-resolution spectra of Ti 2p and Au 4f are presented in Fig. 4c and d, respectively. As displayed in Fig. 4c, two peaks with the binding energy at approximately 458.4 and 464.2 eV can be assigned to Ti 2p_3/2_ and Ti 2p_1/2_ spin–orbit components of Ti^4+^, respectively [39]. Figure 4d shows the Au 4f XPS spectrum with two peaks appeared at 83.6 and 87.4 eV for Au 4f_7/2_ and Au 4f_5/2_ levels, respectively, suggesting that Au species exist as metallic state. The relative negative shift (0.4 eV) of Au 4f_7/2_ peak in comparison of bulk Au (4f_7/2_ at 84.0 eV) can be attributed to the electron transfer from oxygen vacancies of the TiO_2_ to Au, which confirms the strong Au/TiO_2_ interaction [40, 41].

**Fig. 5:**
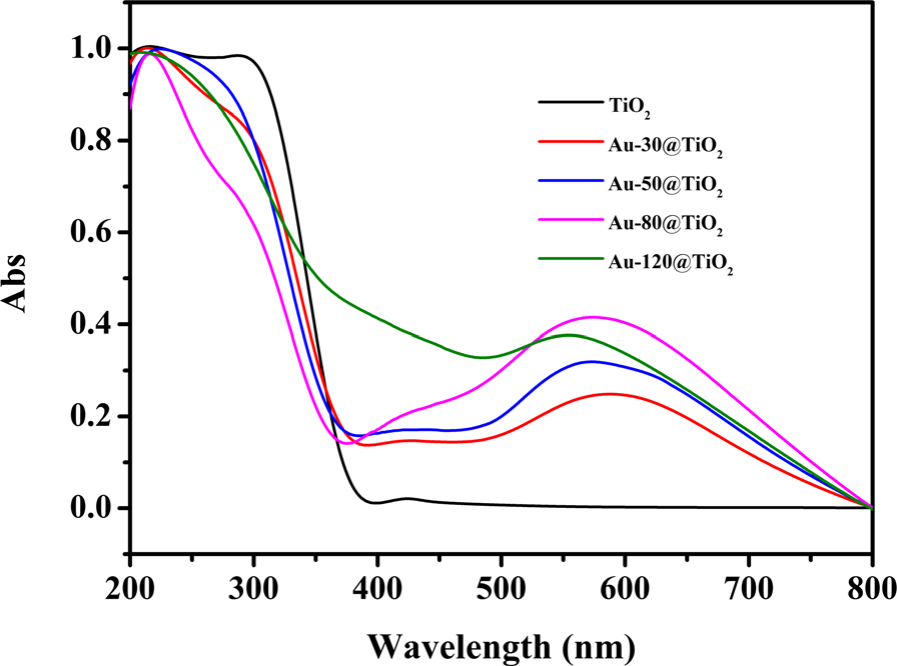
UV–Vis absorption spectra of TiO_2_ and Au-x@TiO_2_

Figure 5 shows the UV–Vis diffuse reflection spectra of the TiO_2_ and Au-x@TiO_2_ nanostructures. An intense absorption band below 400 nm is observed for all these samples, which can be owed to the large band gap of anatase TiO_2_ [42]. Compared with TiO_2_, it can be found that the Au-x@TiO_2_ has not only a similar absorption below 400 nm but also the enhanced absorption range from 400 to 800 nm with a broad absorption peak at about 580 nm arisen from the LSPR effect of Au nanoparticles [43]. These results indicate that a better photocatalytic activity for Au-x@TiO_2_ can be expected under visible light irradiation, especially for the Au-80@TiO_2_ with stronger absorption intensity. The slight shift of the LSPR absorption for Au@TiO_2_ nanostructures with different sputtering time is also reasonable since Au nanoparticle is sensitive to its size and surrounding environment [24, 42]. These observations declare that the Au-x@TiO_2_ photocatalysts can possess a tunable light-harvesting range through adjusting the shape, diameter, and morphology of Au nanoparticles [44].

### Photocatalytic Activity

**Fig. 6:**
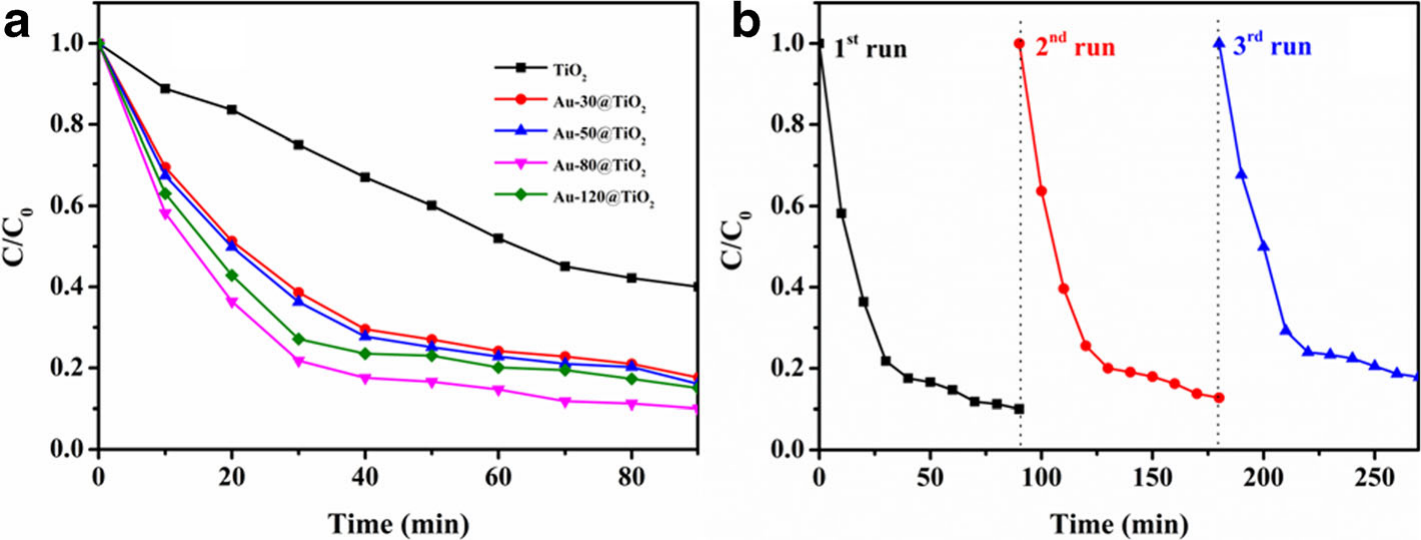
**a** Evaluation of MB concentration versus reaction time in different conditions. **b** Recyclability of the photocatalytic degradation of MB aqueous solution using Au-80@TiO_2_ with three cycles

Removal of organic pollutants from wastewater produced from industry and households has attracted much attention [45–48]. MB is frequently employed as targeted pollutant to evaluate the catalytic efficiency in photocatalytic reactions because the blue color of MB from the absorption at 664 nm would fade gradually with the degradation process [49, 50] and can be easily monitored by UV–Vis absorption spectra. The photocatalytic activities of the TiO_2_ and Au-x@TiO_2_ composites were evaluated by monitoring MB dye’s absorbance at 664 nm to detect the degradation rate under visible light (420 to 780 nm) irradiation. The changes of relative MB concentration versus irradiation time upon the different catalysts are presented in Fig. 6a. For comparison, the photocatalytic activity of pure TiO_2_ nanotubes was first examined. It can be found that about 60% of MB was degraded with TiO_2_ as the photocatalyst under visible light irradiation for 90 min. The relatively low photocatalytic efficiency of TiO_2_ is due to its poor absorption ability of visible light. Compared with the above blank experiment, the Au-x@TiO_2_ photocatalysts exhibit higher degradation efficiency and the degradation efficiency for Au-80@TiO_2_ amounts to about 90% under the same experimental conditions. The promotive photocatalytic properties can be ascribed to increased electron-hole generation rate due to the presence of hetero-interface and the corresponding plasmon-enhanced light absorption [51, 52]. It is known that both high-energy plane (200) of Au and the thickness of TiO_2_ shells are important parameters affecting the activity [24, 53]. Among Au-x@TiO_2_ photocatalysts, with the increased of sputtering time, Au (200) peak exhibits more high-energy planes, as shown in corresponding XRD peak intensity. In addition, Au-120@TiO_2_ with thinner TiO_2_ shell (5 nm) is unable to provide enough reaction sites for the consumption of electrons. Thus, based on the appropriate and similar thickness of TiO_2_ shell over different Au-x@TiO_2_, Au-80@TiO_2_ shows the highest activity.

As heterogeneous catalysts, the reusability of catalyst is also very important in practical application. We performed three consecutive operations to investigate the reusability of the Au-80@TiO_2_. As shown in Fig. 6b, no noticeable deactivation is observed, indicating excellent durability of Au-80@TiO_2_. TEM image of Au-80@TiO_2_ (Additional file 1: Figure S3) after recycling of three times reveals that helical yolk–shell structures of catalysts are well maintained, which further confirms that the confined effect of TiO_2_ nanotubes can prevent Au loss and thus enhances the stability of catalysts.

**Fig. 7:**
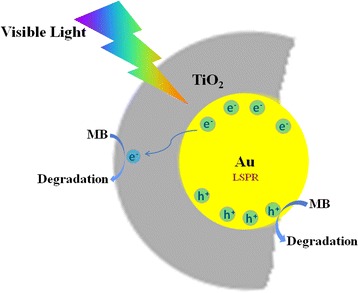
Schematic representation for the mechanism of photocatalytic degradation of MB over Au@TiO_2_

Based on the above results, we propose a photocatalytic process for MB degradation using helical Au@TiO_2_ nanostructures (Fig. 7). Under visible light irradiation, hot electrons are produced by the LSPR effect of Au nanoparticle inside the TiO_2_ nanotube. Subsequent electrons would transfer from Au to the conduction band of TiO_2_. The degradation of adsorbed MB would start from holes (•Au^+^) because the holes can scavenge the surface adsorbed water, generating highly reactive hydroxyl radical species [24, 51, 54]. Simultaneously, the electron injected into the conduction band of TiO_2_ may be trapped by oxygen molecules to form reactive superoxide radicals •O_2_^−^. Then, it can further react with H^+^ to yield active •HO_2_^−^ and •OH radicals. Finally, the organic pollutants may be destroyed by these forming radicals. In this work, it is believed that polarized light rotated by the helical chiral Au@TiO_2_ structure can accelerate the excitation of LSPR, which further enhance the photocatalytic activity of helical Au@TiO_2_. In addition, the adsorbed MB molecule may be excited and transfers an electron to the conduction band of TiO_2_ as the pure TiO_2_ nanotubes show a little photocatalytic activity under visible light irradiation. Thus, the photosensitization effect of MB should also lead to a small part of decomposition of MB.

### SERS activity

**Fig. 8:**
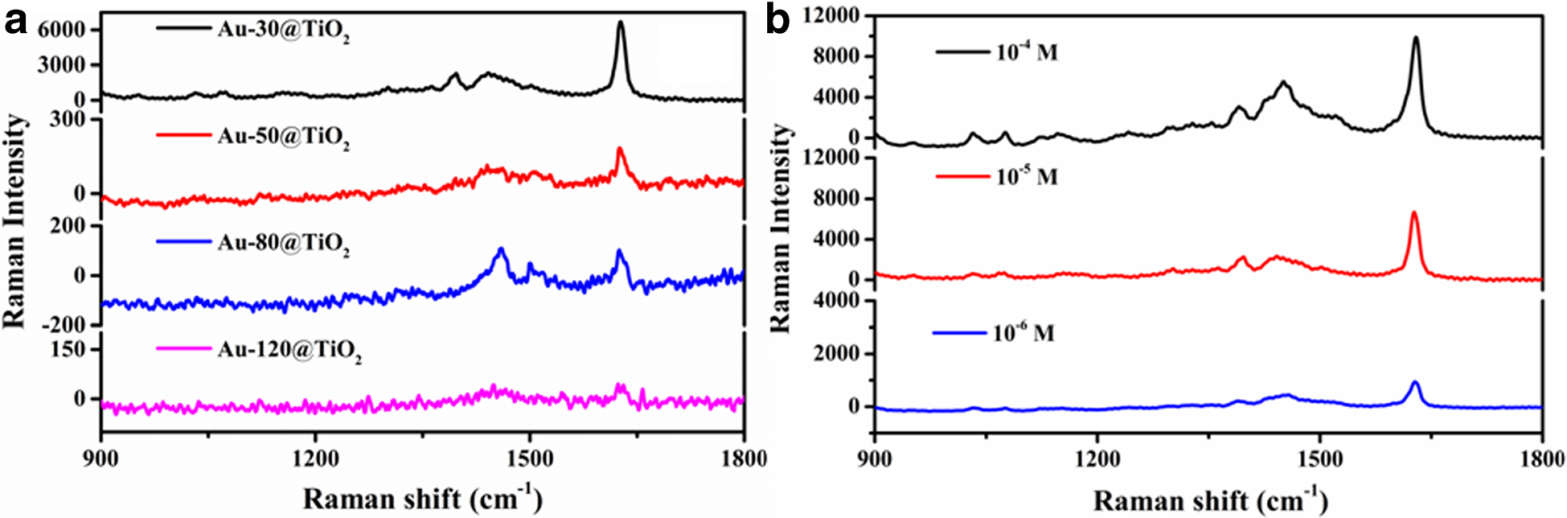
**a** The SERS spectra of 1.0 × 10^−5^ M MB solution collected on the substrates with different Au-x@TiO_2_. **b** The SERS spectra of MB with different concentrations collected on the Au-30@TiO_2_ substrate

To exploit the multifunctional application of such catalysts, we carried out the further experiments by using Au-x@TiO_2_ as SERS substrates to detect the MB molecules adsorbed on the surface of gold nanoparticles. As we can see from Fig. 8a, upon probed with 1.0 × 10^−5^ M MB solution, SERS activity of the as-prepared substrate decreases with the increase of Au sputtering time from 30 to 120 s. This result indicated that Au-30@TiO_2_ has the most excellent SERS performance, implying that Au nanoparticles contacted with TiO_2_ nanoparticles may form a large number of hot spots, which can facilitate to effective SERS enhancement [55]. To explore the influence of varying concentrations of MB solution on the detection ability of Au-30@TiO_2_, Raman measurement was also carried out. As presented in Fig. 8b, the intensity of Raman signal is decreased with the decrease of MB concentrations ranging from 10^−4^ to 10^−6^ M. The discernable Raman signal of 10^−6^ M MB with the Raman band varying from 900 to 1500 cm^−1^, indicating that Au-30@TiO_2_ acted as SERS substrate, can detect the concentrations of MB as low as 10^−6^ M, which shows potential applications for detecting pollutants [56].

## Conclusions

In this study, we have successfully synthesized Au@TiO_2_ yolk–shell heterogeneous nanocomposites with helical coil-like morphology and investigated their multifunctional use including photocatalysis and the SERS effect. The visible photocatalysis degradation of MB displays that the obtained Au-x@TiO_2_ composite with the Au nanoparticles sputtering time of 80 s shows the highest photocatalytic performance because of the increased light absorption and the restriction of the recombination of the photoexcited electron-hole pairs by the LSPR effect of Au nanoparticles. Raman measurements suggest that the Au-x@TiO_2_ can be used as efficient SERS-active substrates. Considering its fascinating properties and features, the novel heterogeneous nanocomposite may provide inspiration in various areas, including water splitting and solar cells. Furthermore, the helical yolk–shell Au@TiO_2_ model system studied here can be extended to the design of other heterostructures, such as Ag@TiO_2_, Au@ZnO, and Au@NiO, for application in solar conversion.

## Additional file

Additional file 1: Supporting information. **Figure S1.** SEM images of CNCs. **Figure S2.** TEM images and the size distribution analysis of Au nanoparticles of (a1 and a2) Au-30@TiO_2_; (b1 and b2) Au-50@TiO_2_; (c1 and c2) Au-80@TiO_2_; (d1 and d2) Au-120@TiO_2_. **Figure S3.** TEM image of the Au-80@TiO_2_ after photocatalytic reaction. (DOC 11017 kb)
