# Supplementary material for: The Preparation of Au@TiO2 Yolk–Shell Nanostructure and its Applications for Degradation and Detection of Methylene Blue
Source: Nanoscale Res Lett. 2017 Sep 18;12:535. doi: 10.1186/s11671-017-2313-4 (PMC5603455; doi:10.1186/s11671-017-2313-4)
Supplement: Additional file 1: — Supporting information. Figure S1. SEM images of CNCs. Figure S2. TEM images and the size distribution analysis of Au nanoparticles of (a1 and a2) Au-30@TiO2; (b1 and b2) Au-50@TiO2; (c1 and c2) Au-80@TiO2; (d1 and d2) Au-120@TiO2. Figure S3. TEM image of the Au-80@TiO2 after photocatalytic reaction. (DOC 11017 kb) [file 11671_2017_2313_MOESM1_ESM.doc]

## Supplementary Material

**The preparation of** **Au@TiO_2_** **yolk-shell nanostructure and its applications for degradation and** **detection of** **methylene blue**

Gengping Wan, Xiange Peng, Min Zeng, Lei Yu, Kan Wang, Xinyue Li, Guizhen Wang*

*Key Laboratory of Advanced Materials of Tropical Island Resources (Hainan University), Ministry of Education, Haikou 570228, P. R. China*

**Corresponding author. Tel.: +86 0898 66268172; Fax: +86 0898 66168037; E-mail address: wangguizhen0@hotmail.com.*


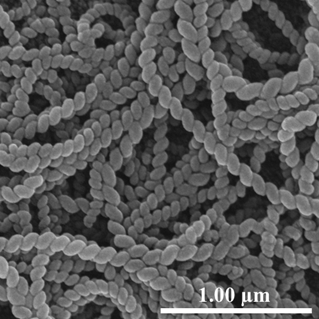


**Figure S1** SEM images of CNCs.


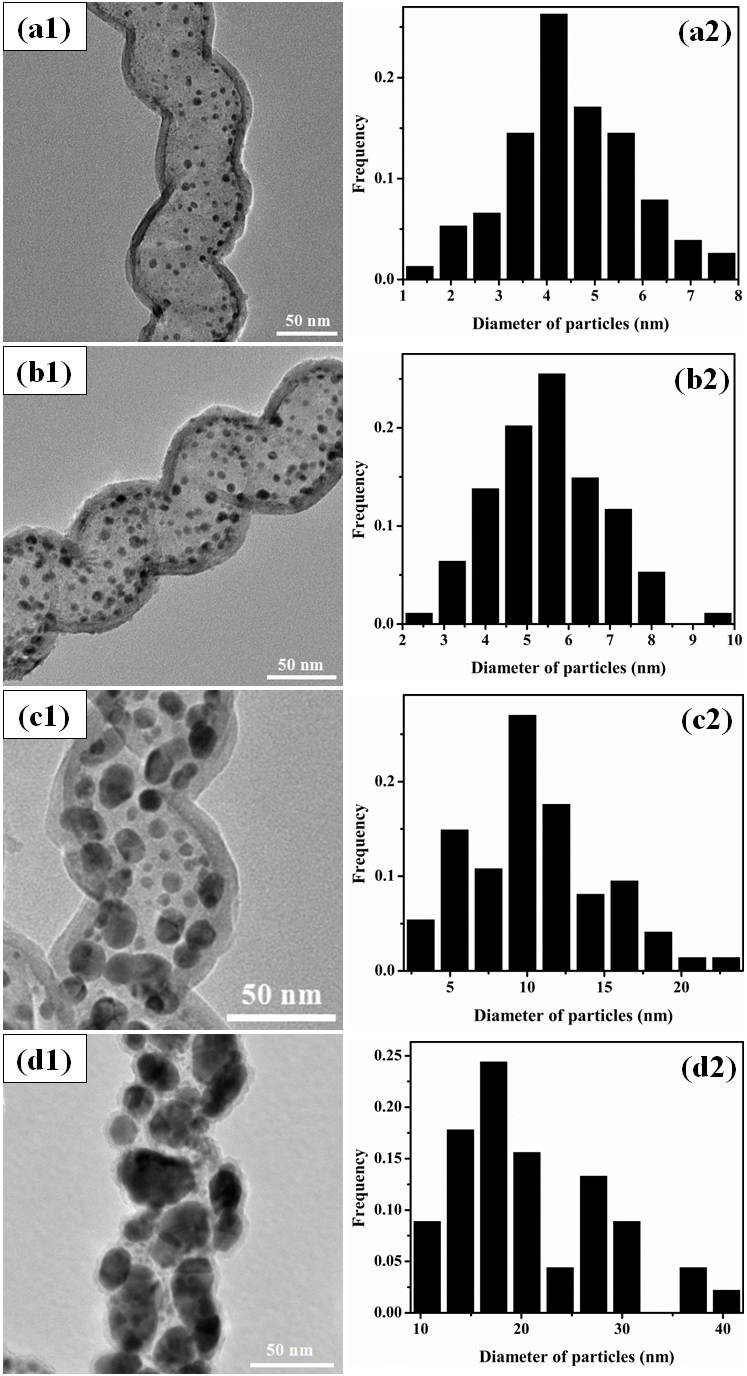


**Figure S2** TEM images and the size distribution analysis of Au nanoparticles of (a1 and a2) Au-30@TiO_2_; (b1 and b2) Au-50@TiO_2_; (c1 and c2) Au-80@TiO_2_; (d1 and d2) Au-120@TiO_2_.


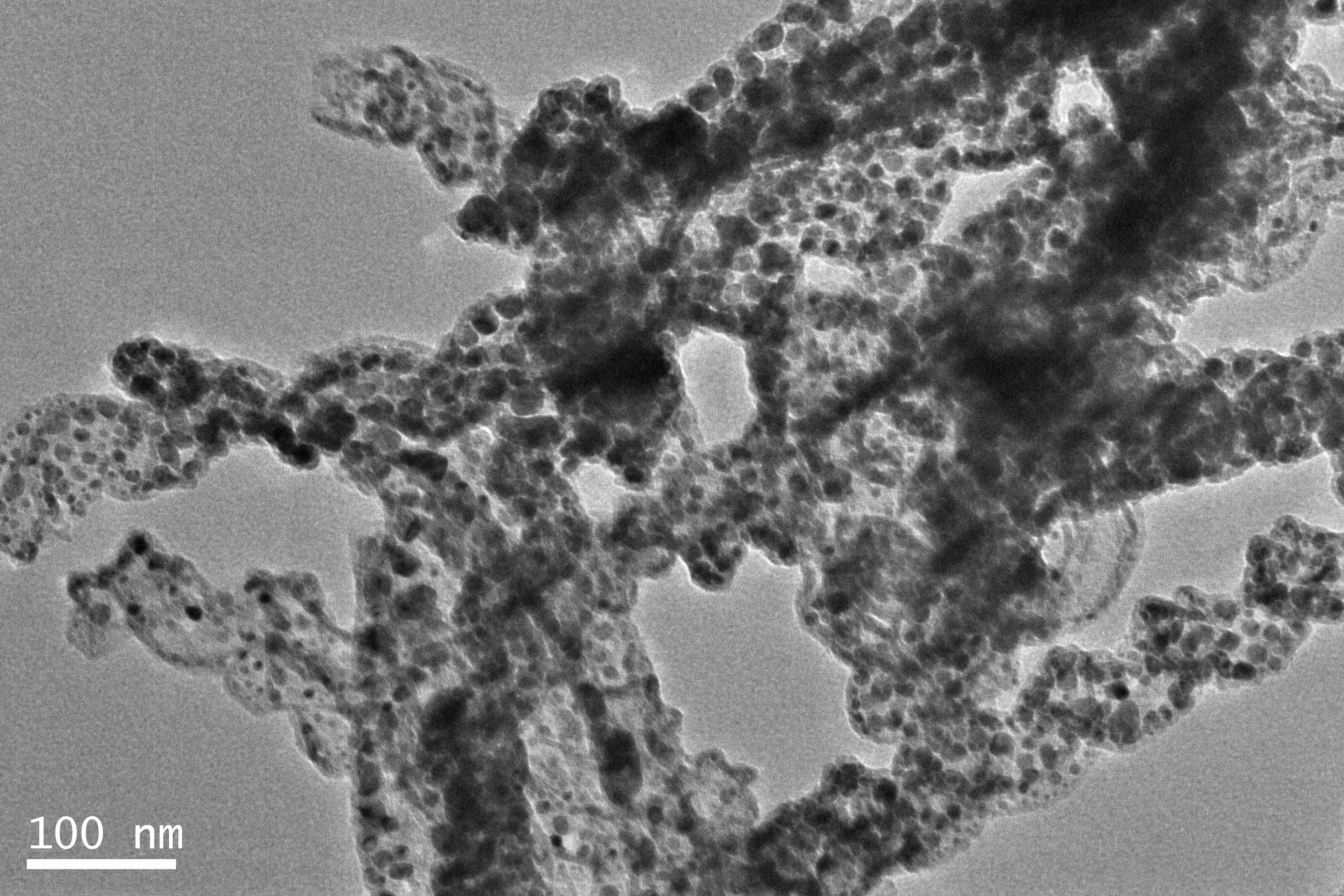


**Figure S3** TEM image of the Au-80@TiO_2_ after photocatalytic reaction**.**
